# Effect of copper oxide (CuO) and vanadium oxide (V_2_O_5_) addition on the structural, optical and electrical properties of corundum (α-Al_2_O_3_)

**DOI:** 10.1038/s41598-023-43309-1

**Published:** 2023-09-26

**Authors:** Mohammed Abdullah Ali Al-Mushaki, Sami Amin Al-Ariki, Adnan Alnehia

**Affiliations:** Department of Physics, Faculty of Applied Science, Thamar University, 87246 Dhamar, Yemen

**Keywords:** Materials science, Nanoscience and technology, Physics

## Abstract

In this work, we prepared a pure α-Al_2_O_3_, α-Al_2_O_3_/CuO (AC) and α-Al_2_O_3_/V_2_O_5_ (AV) nanocomposite. The sol–gel method was used to prepare pure α-Al_2_O_3_, (AC) and (AV) samples at 1200 °C. Structural, electrical, and optical properties of the prepared samples were investigated using the X-ray diffraction (XRD), UV–Visible spectrophotometer, and conductivity meter, respectively. The XRD results confirmed the crystalline nature and the presence of the hexagonal structure of α-Al_2_O_3_, the rhombohedra structure of CuAlO_2_ and the tetragonal structure of V_2_O_5_. Moreover, the crystallite size of pure α-Al_2_O_3_ was 43.1 nm, while the crystallite size of α-Al_2_O_3_ in samples AC and AV nanocompsite was 24.05 nm and 34.84 nm respectively. The optical measurements showed that the band gap α-Al_2_O_3_ decreased significantly from 5.28 eV for pure to 3.7 and 3.4 eV to AC and AV respectively. The DC electrical conductivity (σ_d.c_) values were measured for all prepared samples at room temperature. The electrical conductivity was 2.4 × 10^–7^ and 1.8 × 10^–7^ (Ω cm)^−1^ in AC and AV nanocompsite respectively, while ionic conductivity (σ_ion_) decreased from 3 × 10^–10^ in pure α-Al_2_O_3_ to 7 × 10^–5^ and 1 × 10^–5^ in AC and AV nanocompsite, respectively. The results showed an improvement in the structural, optical, and electrical properties, which may make these materials a candidate for use in many applications, such as photocatalytic, gas sensors, optoelectronics, microelectronics, semiconductor devices, ……etc.

## Introduction

Alumina or aluminum oxide (Al_2_O_3_) is one of the ceramic materials and is used in a wide range of applications such as catalysts, adsorbent, transparent armor for ballistic instrument, discharge lamps, laser, infrared (IR) airborne sensors^1^. There are many forms of Al_2_O_3_ (α, κ, γ, β, θ, χ, δ, ή)^2^. Due to its stable thermodynamics, it is considered α-Alumina Oxide (α-Al_2_O_3_ /corundum) one of the most important phase, α-Al_2_O_3_ has a variety of applications, including ceramic, high-strength materials, transparent armor for ballistic performance, catalysts, catalyst support, adsorbents, and electronic matching like high-performance Field Effect Transistors (FETs), optoelectronics, electrical insulators, thermoluminescent dosimeters, light-emitting display, cutting tools, lasers, spark plugs, and gas sensor^1,3–5^. α-Al_2_O_3_ is formed at temperatures above 1100 °C, with a hexagonal crystalline structure and lattice parameters a = 4.758 Å and c = 12.991 Å^6,7^. (α-Al_2_O_3_) has direct energy transition and energy gap (E_g_) 4.116 eV^5^ 8.8 eV^8^. Vanaduim oxide phases include V_2_O_5_, VO_2_, V_2_O_3_, and multiphase V_x_O_y_. Among all vanadium oxides (V_2_O_5_) is the most stable and has a high oxidation state. Due to their unique structural properties, vanadium oxide-based materials have attracted a lot of attention recently for applications such as solar cells, gas sensors, optical-electrical switches, chemical sensing and electrochromic device optoelectronic devices^9^. Vanadium oxides (V_2_O_5_) has direct energy gap (Eg = 2.2–2.8 eV), an orthorhombic and tetragonal crystalline structure and lattice parameters a = 3.561 Å, b = 11.501 Å, c = 4.378 Å^5,10^. Copper oxide (CuO) is p-type semiconductor with smallest energy gap (Eg = 1.2–1.9) eV, with monoclinic crystalline structure and lattice parameters a = 4.69 Å, b = 3.42 Å, c = 5.13 Å^11^. There are many studies that prepared pure α-Al_2_O_3_ and doped with some metallic elements, but the prepartion of pure α-Al_2_O_3_ as nanocomposite with copper oxide or vanaduim oxide was scarce,and from this point we sought to prepare pure α-Al_2_O_3_, α-,Al_2_O_3_/CuO(AC) and α-Al_2_O_3_/V_2_O_5_ (AV) as a nanocomposite to improve the properties and search for its uses in many other applications. There are many methods used to prepare oxides as pure and nanocomposite materials, such as sol gel^5,6^ hydrothermal^12^, Co_2_ laser vaporization^2^, physical vapro deposition(PVD)^13^….etc. The sol gel technique has been most used because it allows for low temperature synthesis, with excellent purity and simple control of the reaction conditions^6^.

## Experimental details

### Materials

The materials that used in this work include: Aluminum nitrate Al(NO_3_)_3_·9H_2_O (HIMEDIA, 95%), Copper nitrate trihydrate Cu(NO_3_)_2_.3H_2_O (HIMEDIA, 99%) and Ammonium Monovanadate (NH_4_VO_3_) HIMEDIA, 99% and Ethanol C_2_H_5_OH (SEGMA, 96%).

### Experimental procedure

#### Synthesis

#### Synthesis pure α-Al_2_O_3_

To prepare pure α-Al_2_O_3_, 15 g of aluminum nitrate (Al(NO_3_)_3_·9H_2_O) was dissolved in 40 ml of ethanol to obtain a 1 molar solution at room temperature by using magnetic stirrer for 20 min until became solution was homogeneous. Increasing the temperature to 80 °C and moving continuously for 20 min until became the gel by using a magnetic stirrer. The gel stayed in the beaker for 24 h, after that the gel dried in an oven at 180 °C for 2 h. Then grind until it a became soft powder, and put in the oven for 2 h at 1200 °C.

#### Synthesis of samples

To prepare the 0.8Al:0.2Cu (AC) sample, 12 g of aluminum nitrate (Al(NO_3_)_3_·9H_2_O) was dissolved in 40 ml of ethanol to obtain a 0.8 M solution, and 1.933 g copper nitrate trihydrate (Cu(NO_3_)_2_·3H_2_O) was dissolved in 40 ml of ethanol to obtain a 0.1 molar solution. Each solution was stirred separately for 20 min at room temperature until each solution became homogeneous, then all solution were mixed with each other and stirred for 20 min at room temperature until it became homogeneous then stir the homogeneity solution for 20 min at 80 °C until gel formed, the gel stayed in the beaker for 24 h, after that it was dried in the oven at 180 °C for 2 h. Then grind until it a became soft powder. All the samples were put in oven for 2 h at 1200 °C, and left until 24 h for calcinations they were ready for diagnosis. The other samples were prepared in the same way. Also, all samples were made into pellets for electrical measurements. All the pellets were prepared with a pressing machine (Carver) under a pressure of 6000 kg (diameter (d) of pellet is 13 mm and the thickness (L) was 2 mm).

### Characterizations

The structural properties of the samples were investigated by the X-ray diffraction (XRD) technique using XD–2 X-ray diffractometer with CuKα radiation of λ = 0.154056 nm. The optical properties of the samples were investigated using a UV–Vis spectrophotometer (Hitachi U3900 with software of Varian Cary 50). The electrical conductivity measurements of the prepared samples were carried out using (conductivity meter and 3540 PH).

## Results and discussion

### Structure properties

**Figure 1:**
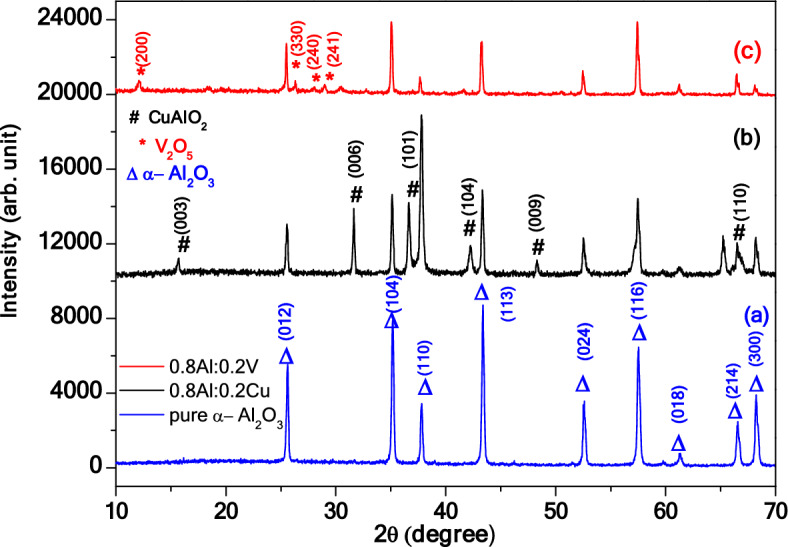
XRD patterns of α-Al2O3, AC and AV nanocompsite.

XRD device was used to determine the crystal structure and crystallite size of the prepared samples. In the first sample XRD patterns of pure α-Al_2_O_3_ was displayed as in Fig. 1a. A number of diffraction peaks of α-Al_2_O_3_, were designated to (012), (104), (110), (113), (024), (116), (018), (214) and (300) planes, which corresponding with 2θ (25.50, 35.20, 37.70, 43.30, 52.60, 57.460, 61.30, 66.50 and 68.20), respectively. The crystalline structure of α-Al_2_O_3_ is hexagonal and space group: R-3c which agrees with the standard (JCPDS card, No. 00-46-1212)^5,7,14–16^. The high intensity of pure α-Al_2_O_3_ for all almost peaks are observed.

In the second sample, XRD patterns showed a formed α-Al_2_O_3_, CuAlO_2_ nanocomposite, as shown in Fig. 1b. The intensity peaks of XRD patterns α-Al_2_O_3_ were reduced, the reason behind the low intensity of α-Al_2_O_3_ due to insert the impurity material of CuO leads to disorder in crystal regulation as well forming deformation for pure α-Al_2_O_3_. The new peaks of CuAlO_2_ were at 2θ values (15.420, 31.50, 36.60, 42.220, 48.320) and (65.40) which are corresponding to (003), (006), (101), (104), (009) and (110) planes, respectively. Agree with standard (JCPDS card, No. 00-035-1401)^15^. The XRD pattern showed that the crystalline structure of CuAlO_2_ was rhombohedra and (space group: R-3m). Occurrence the CuAlO_2_ phase, in the structure due to the eutectic reaction of (Cu^+^ and Cu_2_O) with Al_2_O_3_ as the following^17^:

$$ {\text{2CuO}} + {\text{H}}_{{2}} \mathop{\longrightarrow}\limits^{{{\text{Heated}} }} {\text{Cu}}_{{2}} {\text{O }} + {\text{H}}_{{2}} {\text{O}} $$

$$ {\text{Cu}}_{{2}} {\text{O }} + {\text{Al}}_{{2}} {\text{O}}_{{3}} \mathop{\longrightarrow}\limits^{{{\text{Heated}}}} {\text{2CuAlO}}_{{2}} $$

In the third sample, XRD patterns shown α-Al_2_O_3_, V_2_O_5_ nanocomposite as shown in Fig. 1c, the intenisity peaks of α-Al_2_O_3_ were reduced,the reason behind of low intensity of α-Al_2_O_3_ due to insert the impuity material of V_2_O_5_ which lead to disorder in crystal regulation as well forming deformation for pure α-Al_2_O_3_. The new peaks at 2θ (12.1°, 26.2°, 27.8° and 28.8°) which are corresponding to (200), (330), (240) and (241) planes respectively, of tetragonal structure of V_2_O_5_ card No (JCPDS card, No. 00-45-1074)^18^ were observe.

The average crystallite size (D) of the pure α-Al_2_O_3_, AC and AV nanocomposite were calculated by Debay–Scherrer equation Eq. (1)^19,20^.

1 $$ {\text{D }} = \frac{{0.89{{ \lambda }}}}{{{\beta \text{COS}}\theta }} $$

where λ (0.154 nm) represents the wave length of X-ray, θ indicates Bragg’s angle, and (β): the from full width at half maximum (FWHM). The result were shown as in Table 3. The crystallite size of pure α-Al_2_O_3_ was 43.1 nm. In the (AC) nanocomposite the crystallite size was decrease to 24.05 nm as shown in Table 3. This is decreasing in crystallite size due to that the molar concenteration of Cu^+1^ implying in evolution of secondary phase controls the particle size of the parent phase (α-Al_2_O_3_)^21^. The results which obtained of the crystallite size are in a good agree with^22^. In the (AV) sample, the crystllite size decrease to 34.84 nm, this decrease may be occur due to the ionic radius of the aluminum oxide (0.54 Å) less than ionic radius of vanadium oxide (0.59 Å). Also, the molar concenteration of V^+5^ implying that the evolution of secondary phase controls the particle size of the parent phase (α-Al_2_O_3_), to some extent during crystallization^21^. The results were in a good agree with^23,24^. On the other hand, due to the importance of the dislocation density (δ) in the mechanical and structural properties, it was calculated using Eq. (2)^25^.

2 $$ \delta \, = { 1 }/{\text{ D2}} $$

All the results show in Table 2. In addition, the lattice constants (a, b and c) were calculated _2_ using the Eqs. (3) and (4).

3 $$ \frac{{\text{1}}}{{{\text{d}}^{{\text{2}}} }}{\text{ = }}\frac{{{\text{h}}^{{\text{2}}} }}{{{\text{a}}^{{\text{2}}} }}{\text{ + }}\frac{{{\text{k}}^{{\text{2}}} }}{{{\text{b}}^{{\text{2}}} }}{\text{ + }}\frac{{{\text{l}}^{{\text{2}}} }}{{{\text{c}}^{{\text{2}}} }}  $$

4 $$ \frac{{\text{1}}}{{{\text{d}}^{{\text{2}}} }}{{ = }}\frac{{\text{1}}}{{{\text{sin}}^{{\text{2}}} {{\beta }}}}\left( {\frac{{{\text{h}}^{{\text{2}}} }}{{{\text{a}}^{{\text{2}}} }}{{ + }}\frac{{{\text{k}}^{{\text{2}}} {\text{sin}}^{{\text{2}}} {{\beta }}}}{{{\text{b}}^{{\text{2}}} }}{{ + }}\frac{{{\text{l}}^{{\text{2}}} }}{{{\text{c}}^{{\text{2}}} }} - \frac{{{{{\text 2hl cos}\beta }}}}{{{\text{ac}}}}} \right) $$

The calculated lattice constants were in good agreement with the last studies^22^. The unit cell volume (V) and The strain (ε) calculated using the Eqs. (5)–(7)^26^, respectively. The results are shown in Tables 1 and 2.

5 $$ {\text{V}} = \, 0.{\text{866\,\,a}}^{2}\, {\text{c}} $$

6 $$ {\text{V}} = {{ \text abc\,\, sin }\beta} $$

7 $$ {\varepsilon  } = \frac{{{{ \beta {\text cos}\theta }}}}{4} $$

**Table 1:** Structure properties of the pure α-Al_2_O_3_, AC and AV nanocomposite.

Samples	Sample code	Phase oxide	2θ (°)	hkl	FWHM (°)	d (Ǻ)		a (Ǻ)	b (Ǻ)	c (Ǻ)
						Expt	Std			
Pure	Pure α-Al_2_O_3_	α-Al_2_O_3_	43.3	113	0.198	2.0852	2.0853	4.756	4.756	12.993
0.8Al:0.2Cu	AC	α-Al_2_O_3_	37.74	110	0.349	2.3817	4.212	4.116	4.212	13.031
		CuAlO_2_	36.6	101	0.332	2.4532	2.4474	2.857	2.857	16.945
0.8Al:0.2V	AV	α-Al_2_O_3_	57	116	0.26	1.6016	1.6014	4.1166	4.1166	13.0212
		V_2_O_5_	27.8	240	0.381	3.202	3.193	14.259	12.295	12.576

**Table 2:** Structure properties of the pure α-Al_2_O_3_, AC and AV nanocomposite.

Samples	Sample code	Phase oxide	C/a	V (A^3^)	D (nm)	δ*10^15^ m^−2^	Strain (ɛ)
Pure	Pure α-Al_2_O_3_	α-Al_2_O_3_	2.731918	254.5136	43.10	5.383261	0.001192
0.8Al:0.2Cu	AC	α-Al_2_O_3_	3.165525	191.2404	24.05	1.7289	0.001214
		CuAlO_2_	5.931047	119.7788	25.2	1.574704	0.001218
0.8Al:0.2V	AV	α-Al_2_O_3_	3.163096	191.0936	34.84	8.237295	0.001127
		V_2_O_5_	0.8819	2556.94	21.47	2.267573	0.092257

### Optical properties

#### Transmission

**Figure 2:**
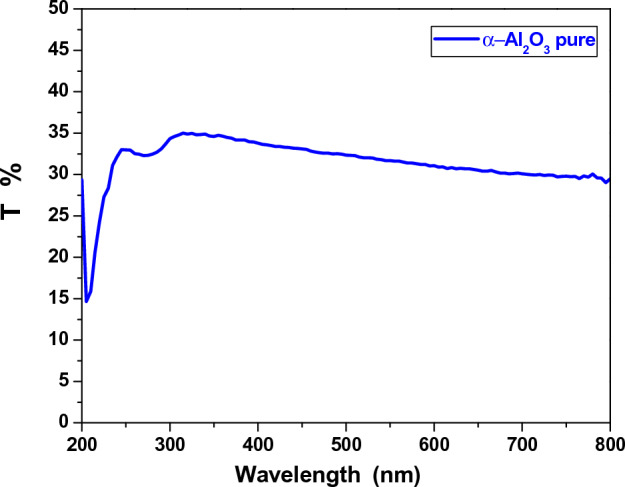
The transmission of pure α-Al_2_O_3_.

**Figure 3:**
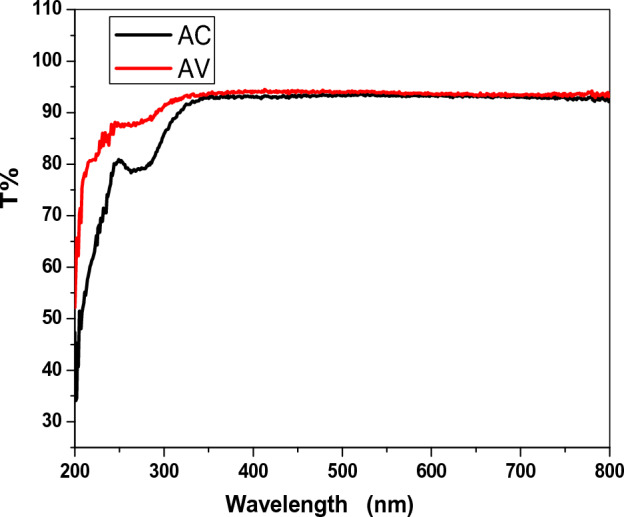
The transmission of AC and AV nanocomposite.

The transmittance spectra of pure α-Al_2_O_3_, AC and AV nanocomposite was measured in the range 200–800 nm. The transmittance of pure α-Al_2_O_3_ nanoparticles decreased as the wavelength increase, with the highest increased transmittance occurring at a wavelength of 320 nm, as shown in Fig. 2. The transmittance of AC and AV nanocomposite also decreased, as the wavelength increased, with transmittance values of 92% and 94%, respectively, as shown in Fig. 3. The increase in transmittance in the sample with added copper and vanadium can be attributed to the formation of new energy levels within the band gap of the α-Al_2_O_3_ crystal lattice. When copper and vanadium ions are added to the α-Al_2_O_3_ lattice, they introduce new energy levels that allow for the absorbed of light that allow for the absorbed by the crystal lattic, leading to an increase in transmittance. The exact mechanism behind this phenomenon is complex and depends on the specific properties of the added ions and their interaction with the α-Al_2_O_3_ lattice. Ho wever, it is clear that the addition of copper and vanadium ions to the α-Al_2_O_3_ crystal lattice can significantly alter its optical properties, leading to increased transmittance^16^.

#### Absorption

**Figure 4:**
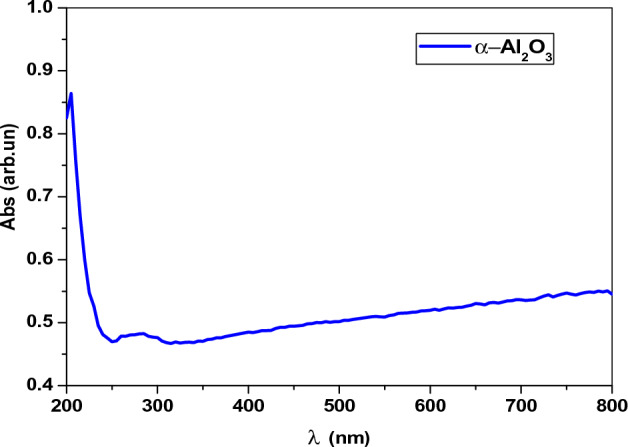
The absorption of pure α-Al_2_O_3_.

**Figure 5:**
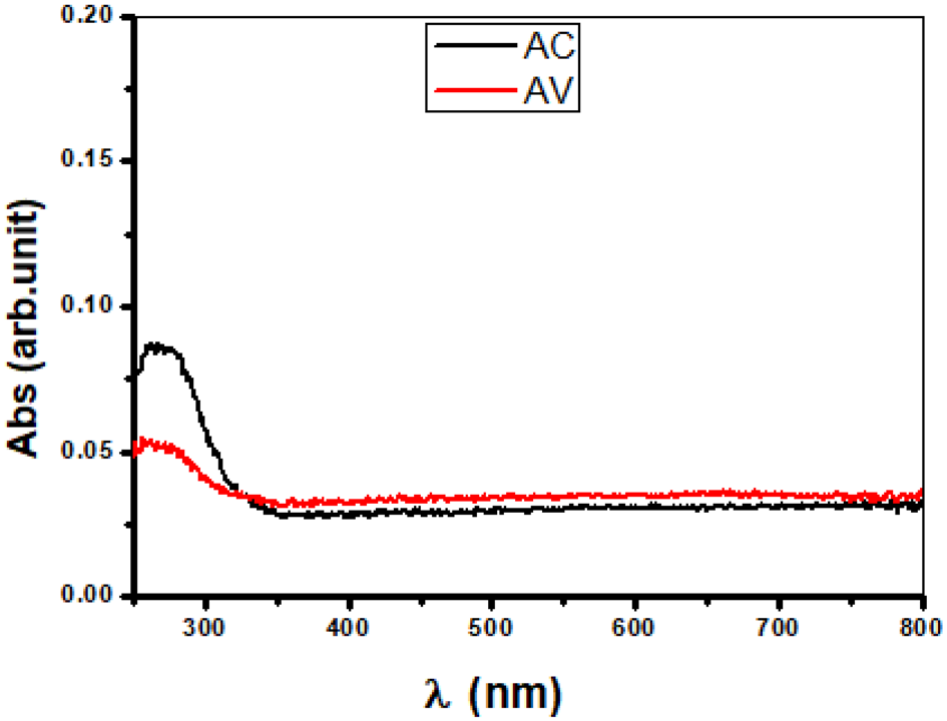
The absorption of AC and AV nanocomposite.

The optical absorpation of the samples were determined at room temperature using the UV–visible spectrophotometer within wavelenghth rang of 200–800 nm. Figure 4 shows the relationship between the absorpation on the Y-axis and the wavelength on the X-axis of pure α-Al_2_O_3_, the highest point of the absorpation was at 204 nm, while the lowest value was at 310 nm, then the absorpation was increase slightly with increasing the wavelength. The wavelength absorption of pure α-Al_2_O_3_ are observed at 280 nm^16^. The absorption of the AC and AV nanocomposites shown as in Fig. 5. Inspectra the absorption edges are observed in the UV–Vis region as 314.8–344 nm for AC and AV nanocomposite, respectively. In these samples absorption bands are attributed to the photoexcitation of electrons from the valence band to the conduction band. Further, the absorption bands are ascribed to the electronic transitions from occupied 2p bands ofoxygen to unoccupied 3d bands of copper and vanadium^27^.

##### Optical band gap energy (E_g_)

**Figure 6:**
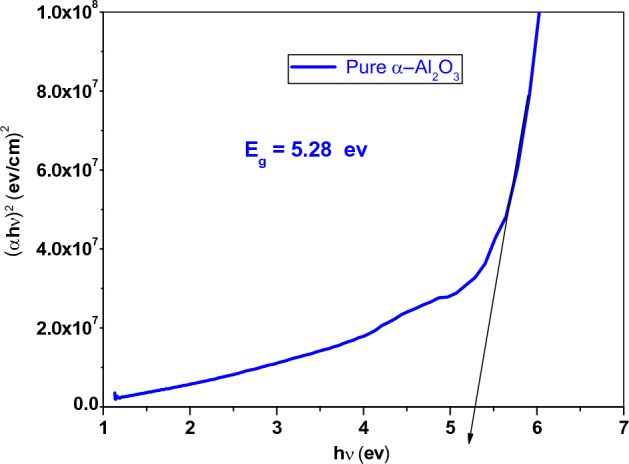
The energy gap of pure α-Al_2_O_3_.

**Figure 7:**
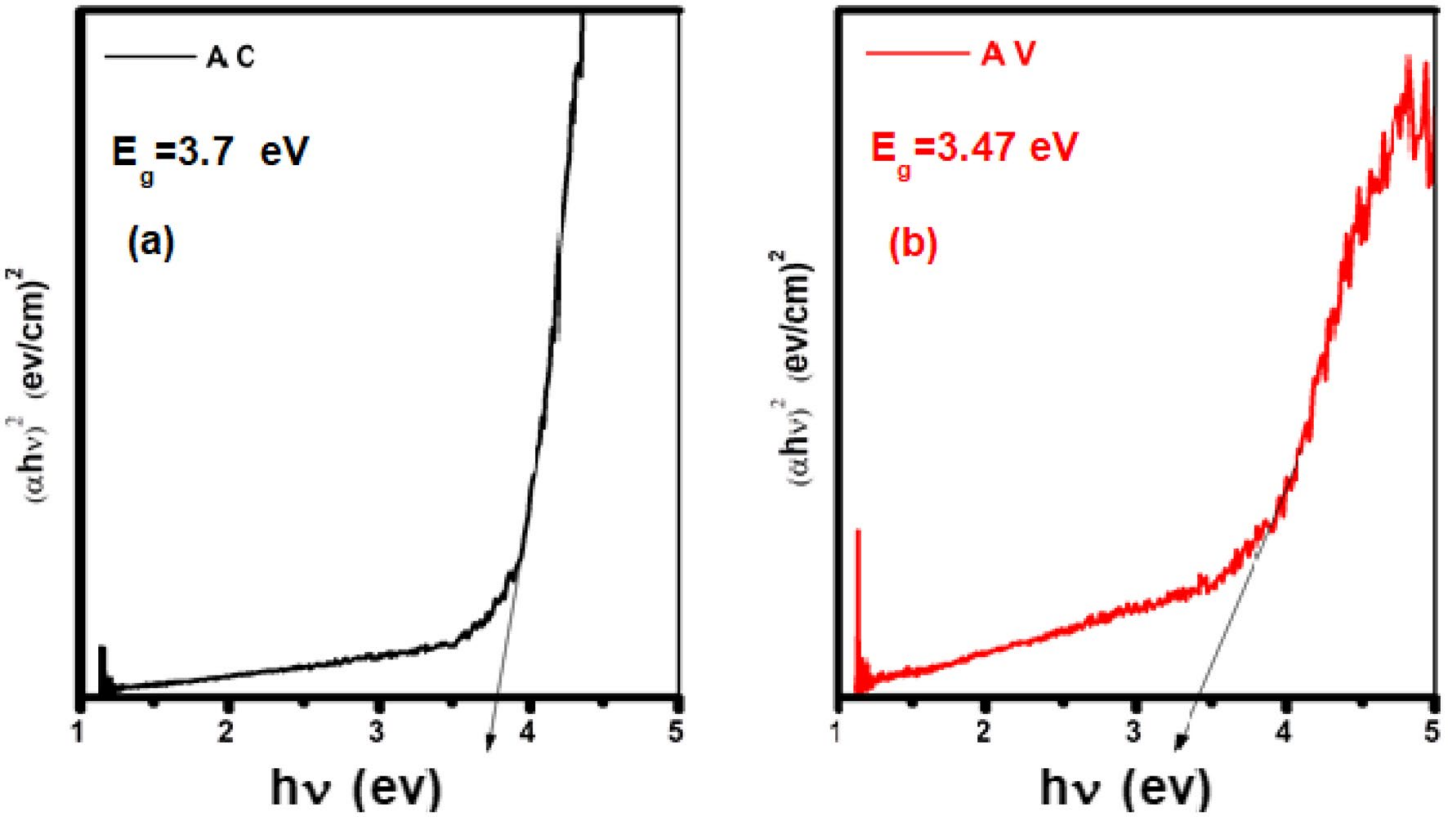
The energy gap of (**a**) AC nanocomposite and (**b**) AV nanocomposite.

The energy gap (E_g_) was calculated by using the following equation^20,28–30^:

8 $$ \alpha \user2{h\nu } = {\varvec{c}}_{1} \left( {\user2{h\nu } - {\varvec{E}}_{{\varvec{g}}} } \right)^{\frac{1}{2}} \user2{ } $$

where C_1_ is a constant, h is the Planck constant and α is the optical absorption coefficient. The energy gap of pure α-Al_2_O_3_ was 5.28 eV as shown in Fig. 6. This result agree with^16^. While the energy gap of AC and AV were 3.7 and 3.47 eV, respectively, as shown in Fig. 7a and b. The addition of 20% Cu^+1^ and 20% V^+1^, reduced the band gap in the nanocomposite. The decrease in the band gap value can be attributed to the appearance of the empty levels induced by defects located in the band gap^5^. It is a well-known that the band gap of any material is influenced by the concentration of defects. In α-Al_2_O_3_, both donor (oxygen vacancies) and acceptor defects (Al interstitials) create energy levels below the conduction band and above the valence band, respectively. The creation of energy levels can be explained by the Frenkel reaction for Al interstitial defects and the Schottky reaction for the oxygen vacancy defects^31^.

### Electrical properties

#### Current-voltag (I–V) measurments

The ohmic resistance (**R**) of pure α-Al_2_O_3,_ AC and AV nanocomposites were calculated from the I–V curve according to ohm law Eq. (9) (ohm law)^32^.

9 $$ R = \frac{V}{I} $$

where (I) is the current and the (V) is the voltag. The electrical conductivity (σ_d.c_) was calculated by using Eq. (10)^33^.

10 $$ {\upsigma }_{d.c} = \frac{L}{RA} $$

where L (Thiknecess), d (Diameter), r (Radius; r = d/2) and A (Area; A = л r^2^ ). The conductivity of material is determined by the presence of free charge carriers, such as electrons or ions, that can move freely within the material^34^. In the case of pure α-Al_2_O_3_, there are no free charge carriers available, resulting in zero conductivity. When copper nitrate (Cu(NO3)2) is added to α-Al_2_O_3_, it introduces copper ions (Cu_2_^+^) into the material. These copper ions can act as charge carriers and contribute to the conductivity of the material^35^. However, the conductivity is still weak because the conductivity is still weak because the concentration of copper ions is relatively low. Similarly, when copper nitrate NH_4_VO_3_ is added to α-Al_2_O_3_, it introduces vanadium ions (V_5_^+^) into the material. These copper ions can act as charge carriers and contribute to the conductivity. However, like with copper nitrate, the conductivity of vanaduim ions is relatively low, resulting in weak conductivity. It is important to note that both copperr and vanaduim are transition metals with partially filled d-orbitals in their electronic configurations. This allows them to easily donate or accept electrons and participate in charge transport within a mateial. The value obtained in this work for electrical conductivity(σ_d.c_) is in agree with conventional value of σ_d.c_ of semiconductors (10^4^–10^−9^ Ω^−1^ cm^−1^)^36^, also is consistent with the average value of α-Al_2_O_3_ (6.87 × 10^–12^ ± 1.22 × 10^–14^ Ω^−1^ cm^−1^)^37^, CuO (1.1 × 10^−4^ and 2.77 × 10^–4^ Ω^−1^ cm^−1^)^38^ and V_2_O_5_ (2.53 × 10^–4^ Ω^−1^ cm^−1^)^39^, (2.48 × 10^−6^ and 6.16 × 10^−8^ Ω^−1^ cm^−1^)^40^.

#### Ionic conductivity (σ_ion_)

The ionic conductivity σ_ion_ of the elctrolyte was measured at room temperture.The ionic conductivity was found to be greater than the electrical conductivity, and this increase may be attributed to the contribution of charged carriers in the liquid, as shown in Table 3.

**Table 3:** Value R, σd.c and σion of pure α-Al_2_O_3_, AC and AV nanocomposite.

Sample	Sample code	L (cm)	d (cm)	r (cm)	A (cm)^2^	R (kΩ)	σ_d.c_ (Ω cm)^−1^	σ_ion_
Pure	Pure α-Al_2_O_3_	0.2	1.3	0.65	1.33	–	–	3E−10
0.8Al:0.2Cu	AC	0.2	1.3	0.65	1.33	588	2.44951E−07	7E−05
0.8Al:0.2V	AV	0.2	1.3	0.65	0.65	769.3	1.87297E−07	1E−05

## Conclusions

In the summary, pure α-Al_2_O_3_, (AC) and (AV) nanocomposite were prepared using Sol–Gel method at 1200 °C. X-ray diffraction showed, the high crystallinity of all samples. The crystallite size dimension was calculated from diffraction data using the formula Debye–Scherrer. The results showed that the crystallite size (D) of pure α-Al_2_O_3_ was 43.1 nm with hexagonal structural, the crystal size of α-Al_2_O_3_ in AC nanocomposite was 24.05 nm, the crystal size of V_2_O_5_ was 21.47 nm with tetragonal structure and the crystal size of CuAlO_2_ was 25.2 nm with rhombohedra structure. The band gab of pure α-Al_2_O_3_ was 5.28 eV, while the band gap of AC and AV nanocomposite were 3.7 and 3.47 eV respectively. The resistance was decreasing with addition concentration of Cu^+1^ and V^+5^.

## Data Availability

The authors confirm that the data supporting the findings of this study are available within the article.
